# A putative role for homocysteine in the pathophysiology of acute bacterial meningitis in children

**DOI:** 10.1186/1472-6890-14-43

**Published:** 2014-11-22

**Authors:** Roney Santos Coimbra, Bruno Frederico Aguilar Calegare, Talitah Michel Sanchez Candiani, Vânia D’Almeida

**Affiliations:** Biosystems Informatics, Research Center Rene Rachou, FIOCRUZ, Av. Augusto de Lima 1715, Belo Horizonte, MG Zip Code: 30190-002 Brazil; Department of Psychobiology, Universidade Federal de São Paulo (UNIFESP/EPM), São Paulo, SP Brazil; Children’s Hospital João Paulo II – FHEMIG, Belo Horizonte, MG Brazil

**Keywords:** Acute bacterial meningitis, Homocysteine, Neurotoxicity

## Abstract

**Background:**

Acute bacterial meningitis frequently causes cortical and hippocampal neuron loss leading to permanent neurological sequelae. Neuron death in acute bacterial meningitis involves the excessive activation of NMDA receptors and p53-mediated apoptosis, and the latter is triggered by the depletion of NAD + and ATP cellular stores by the DNA repair enzyme poly(ADP-ribose) polymerase. This enzyme is activated during acute bacterial meningitis in response to DNA damage induced, on its turn, by reactive oxygen and nitrogen species. An excess of homocysteine can also induce this cascade of events in hippocampal neurons.

The present work aimed at investigating the possible involvement of homocysteine in the pathophysiology of meningitis by comparing its concentrations in cerebrospinal fluid (CSF) samples from children with viral or acute bacterial meningitis, and control individuals.

**Methods:**

Homocysteine and cysteine concentrations were assessed by high-performance liquid chromatography in CSF samples from nine patients with acute bacterial meningitis, 13 patients with viral meningitis and 18 controls (median age: 4 years-old; range: <1 to 13) collected by lumbar puncture at admission at the Children's Hospital Joao Paulo II - FHEMIG, from January 2010 to November 2011.

**Results:**

We found that homocysteine accumulates up to neurotoxic levels within the central nervous system of patients with acute bacterial meningitis, but not in those with viral meningitis or control individuals. No correlation was found between homocysteine and cysteine concentrations and the cerebrospinal fluid standard cytochemical parameters.

**Conclusions:**

Our results suggest that HCY is produced intrathecally in response to acute bacterial meningitis and accumulates within the central nervous system reaching potentially neurotoxic levels. This is the first work to propose a role for HCY in the pathophysiology of brain damage associated with acute bacterial meningitis.

## Background

Meningitis is the inflammation of the meninges, the protective membranes covering the brain and the spinal cord. According to the etiology, meningitis is classified as viral (VM), also called aseptic meningitis, or bacterial (BM). VM is mainly caused by enteroviruses and is considered to be typically benign, with an auto-limited course being rarely associated to bad prognostic. As opposite, acute BM is ranked among the ten leading causes of death by infectious diseases worldwide [1]. The main etiological agents of BM are *Streptococcus pneumoniae*, *Neisseria meningitidis*, and *Haemophilus influenzae* type b. Despite significant advances in antimicrobial and intensive care therapies in the last decades, mortality rates associated with BM remain as high as 30%, and among the patients who survive the infection, 30 to 50% have permanent neurological sequelae which include deafness, sensory-motor deficits, seizure disorders, cerebral palsy, mental retardation, and learning impairment associated to neuronal injury [2].

The neurological sequelae associated to meningitis are mainly due to neuron loss by necrosis in the cerebral cortex, and by apoptosis in the hippocampal dentate granule cells [3–5]. The cascade of events that triggers neuronal apoptosis during BM involves the excessive activation of the DNA repair enzyme poly(ADP-ribose) polymerase (PARP) which synthesizes ADP-ribose polymers in response to DNA damage [6]. This process comes at a very high-energy cost depleting NAD + and ATP and thereby causing cell death. PARP may provide a linkage between oxidative DNA damage and apoptosis or necrosis during BM depending upon the severity of the ATP and NAD + withdrawal. Homocysteine (HCY) is a sulfur amino acid produced from methionine by a methylation cycle [7]. HCY induces neuronal apoptotic death by over stimulating N-methyl-D-aspartate (NMDA) receptors [8, 9] and by enhancing the production of free radicals [10, 11], two hallmarks of the pathophysiology of acute BM [12–14]. Furthermore, elevated HCY levels in the nucleus of cells may induce DNA strand breaks by disturbing the DNA methylation cycle [15]. Consistent with this hypothesis, Kruman et al. [16] have shown a major role for PARP activation in HCY-induced neuronal apoptosis and increased neuronal vulnerability to excitotoxicity.

Thus, this study aims to investigate the possible involvement of HCY in the pathophysiology of meningitis by comparing its levels and those of cysteine (CYS), another sulfur amino acid two steps downstream to HCY in the same pathway, in cerebrospinal fluid (CSF) samples from infant patients with acute BM, VM, and control children.

## Methods

HCY and CYS levels were assessed by high-performance liquid chromatography (HPLC) in CSF samples collected by lumbar puncture from 40 children (median age: 4 years-old; range: <1 to 13 years) at admission at the Children’s Hospital João Paulo II – FHEMIG, Belo Horizonte, Brazil, with suspected meningitis from January 2010 to November 2011. The cohort comprised: a) nine patients with acute BM confirmed by CSF culture and/or latex agglutination test, being six infected with pneumococci and three with meningococci; b) 13 patients with VM, who had clinical signs of meningitis but presented normal or slightly altered cytochemical parameters in CSF, and negative CSF and blood latex and culture for bacterial pathogens [1]; c) 18 controls subjects attending the hospital because of a suspect of meningitis, but who had no infection of the central nervous system (CNS) or neurodegenerative diseases at the definitive diagnostic. The standard CSF cytochemical parameters assessed for diagnostic purpose immediately after puncture were protein and glucose concentrations, white blood cell count and percentage of polymorphonuclear neutrophils [17]. Patients previously treated with antibiotics, or whose CSF samples contained more than 50 erythrocytes per mm^3^, indicating blood contamination due to puncture accident, were excluded. All patients included in this study survived meningitis, but one children who had pneumococcal meningitis developed total hearing loss in one ear. An aliquot of the CSF sample was centrifuged at 5.000 rpm for 10 minutes, at 4°C, and the supernatant was stored at -80°C.

HCY and CYS concentrations in CSF samples were measured by high-performance liquid chromatography (HPLC) with fluorimetric detection after derivatization of sulfur amino acids with fluorescent 7-fluorobenzofurazan-4-sulfonic acid as previously published [18]. Calibration curves were linear up to 200 μM and 800 μM for homocysteine and cysteine, respectively. The limit of detection for homocysteine was 0.16 μM. For statistical analysis, HCY and CYS concentrations below the detection limit of the method were arbitrarily assigned to zero.

The variances of medians of all groups were compared using the Kruskal-Wallis test and, subsequently, pairs of groups were compared using the Dunn’s test. Correlations were tested using the Spearman’s test. The threshold for statistical significance was p <0.05. These statistical analyses were performed with the package GraphPad Prism 5.01 (GraphPad Software, Inc., La Jolla, CA, USA).

This study was approved by the Brazilian Committee for Ethical Research (process number: 25000.199054/2008-18). The guardians of all patients were informed about the study and signed a consent form.

## Results and discussion

The HCY and CYS medians of the three groups varied significantly (p <0.005). HCY and CYS concentrations were increased in the CSF of patients with BM, regardless of the pathogen when compared to controls or patients with VM (Table 1). The median concentration of HCY in the CSF of patients with BM (0.69 μM) was higher than the lowest concentration reported to induce apoptosis in cultured hippocampal neurons (0.5 μM) [16]. In addition, for one patient with BM, the HCY and CYS levels were measured in CSF samples collected at admission during the acute infection and after cure (HCY: 0.690 vs. 0 μM; CYS: 51.231 vs. 0 μM). These results indicate that the HCY and CYS levels were increased during acute BM, dropping to normal levels after cure. No correlations were found between HCY or CYS levels and the standard CSF cytochemical parameters.

**Table 1:** **Cytochemical parameters in the CSF of patients with meningitis and controls**

	BM		VM		Ctrl	
	(n =9)		(n =13)		(n =18)	
	Mean ± SD	Median	Mean ± SD	Median	Mean ± SD	Median
	(range)		(range)		(range)	
HCY (μM)	1.08 ± 1.43	0.69*^†^	0	0*	0.05 ± 0.16	0^†^
	(0.0 – 4.37)		(-)		(0.0 – 0.52)	
CYS (μM)	30.77 ± 18.46	29.34*^†^	8.41 ± 5.49	8.19*	7.72 ± 4.70	8.33^†^
	(13.37 – 67.64)		(0.0 – 19.92)		(0.0 – 16.48)	
% PMN	77.89 ± 27.86	86*	35.92 ± 37.38	19	16.22 ± 26.43	3.5*
	(5 – 95)		|(0 – 95)			
WBC /μL	1383 ± 2131	120*	75.38 ± 92.96	50^†^	4.05 ± 4.10	2.5*^†^
	(18 – 5600)		(8 – 348)		(1 – 9)	
Protein (mg/dL)	157.83 ± 136.1	117*^†^	32.67 ± 12.49	26*	33.34 ± 18.98	26.15^†^
	(35 – 441)		(20 – 56.6)		(20 – 85)	
Glucose (mg/dL)	48 ± 15.67	48	53 ± 19.62	57.5	60.94 ± 9.59	60
	(20 – 71)		(40 – 72)		(50 – 84)	

BM = bacterial meningitis; VM = viral meningitis; Ctrl = controls; HCY = homocysteine; CYS = cysteine; WBC = white blood cell; PMN = polymorphonuclear neutrophils; SD = standard deviation; * or ^†^ = p <0.05.

This is the first work reporting the association between increased HCY levels within the CNS and the pathophysiology of acute BM. The specific increase in HCY concentrations in the CSF of children with acute BM, and the absence of correlation between HCY and CYS or the standard CSF cytochemical parameters indicate that the local production of HCY in the intrathecal space is part of the host response to the pneumococci or meningococci invasion of the CNS. The intrathecal accumulation of HCY at potentially neurotoxic levels [16] strongly support the hypothesis that this sulphur amino acid may play a pivotal role in the pathophysiological processes that lead to neuron death and brain damage associated with acute BM. In agreement with these conclusions, Qureshi *et al.* [19] have previously reported increased HCY levels in CSF samples of adults with tuberculous meningitis compared to those with aseptic meningitis or controls. However, these authors did not investigate HCY levels in children with acute BM. The HCY concentrations in adult patients with aseptic meningitis and control adults reported by Qureshi *et al.* were higher than those reported herein for children. This may be explained by the differences in the average age of the patients enrolled in these two studies. Indeed, previously reported reference values for HCY in the CSF from healthy adults ranged from 1.28 to 0.66 μM [20], while for healthy children these reference values were lower than 0,10 μM [21].

## Conclusions

The results presented herein, in the light of the current biomedical literature, suggest that the excess of homocysteine within the central nervous system may play a pivotal role in the pathophysiology of acute bacterial meningitis. Further investigations with larger cohorts and additional studies using animal models are still needed in order to assess the association between increased HCY levels within the CNS and the development of brain damage and permanent neurological sequelae after acute BM. If this association can be definitely established, new adjuvant therapies to prevent brain damage in BM will be conceivable targeting key enzymes of the homocysteine pathway.

## Abbreviations

BM: Bacterial meningitis
CNS: Central nervous system
CSF: Cerebrospinal fluid
CYS: Cysteine
HCY: Homocysteine
HPLC: High-performance liquid chromatography
NMDA: N-methyl-D-aspartate
PARP: Poly(ADP-ribose) polymerase
VM: Viral meningitis.
